# Which client characteristics predict home‐care needs? Results of a survey study among Dutch home‐care nurses

**DOI:** 10.1111/hsc.12611

**Published:** 2018-07-19

**Authors:** Anne O. E. van den Bulck, Silke F. Metzelthin, Arianne M. J. Elissen, Marianne C. Stadlander, Jaap E. Stam, Gia Wallinga, Dirk Ruwaard

**Affiliations:** ^1^ Faculty of Health, Medicine and Life Sciences, Care and Public Health Research Institute (CAPHRI), Department of Health Services Research Maastricht University Maastricht The Netherlands; ^2^ Dutch Healthcare Authority (NZa) Utrecht The Netherlands; ^3^ Dutch Nurses Association (V&VN) District Nurses and Public Health Nurses Utrecht The Netherlands

**Keywords:** casemix, client characteristics, clients’ needs, funding, home care, nurse perspective

## Abstract

Fee‐for‐service, funding care on an hourly rate basis, creates an incentive for home‐care providers to deliver high amounts of care. Under casemix funding, in contrast, clients are allocated—based on their characteristics—to homogenous, hierarchical groups, which are subsequently funded to promote more effective and efficient care. The first step in developing a casemix model is to understand which client characteristics are potential predictors of home‐care needs. Nurses working in home care (i.e. home‐care nurses) have a good insight into clients’ home‐care needs. This study was conducted in co‐operation with the Dutch Nurses’ Association and the Dutch Healthcare Authority. Based on international literature, 35 client characteristics were identified as potential predictors of home‐care needs. In an online survey (May, 2017), Dutch home‐care nurses were asked to score these characteristics on relevance, using a 9‐point Likert scale. They were subsequently asked to identify the top five client characteristics. Data were analysed using descriptive statistics. The survey was completed by 1,007 home‐care nurses. Consensus on relevance was achieved for 15 client characteristics, with “terminal phase” being scored most relevant, and “sex” being scored as the least relevant. Relevance of the remaining 20 characteristics was uncertain. Additionally, based on the ranking, “ADL functioning” was ranked as most relevant. According to home‐care nurses, both biomedical and psychosocial client characteristics need to be taken into account when predicting home‐care needs. Collaboration between clinical practice, policy development, and science is necessary to realise a funding model, to work towards the Triple Aim (improved health, better care experience, and lower costs).

**What is known about this topic**

- Home‐care funding on a fee‐for‐service basis can create perverse volume incentives for providers.
- Casemix funding could potentially stimulate more needs‐based, high‐quality and efficient home care.
- Home‐care nurses have knowledge on determinants of clients’ home‐care needs, which is vital information for developing a casemix model.

**What this paper adds**

- According to current casemix models and relevant literature, 35 client characteristics are potential predictors of home‐care needs.
- Home‐care nurses participating in this study agree on the relevance of 15 of these characteristics, including “physical functions” and “social support.”
- This study’s results can be used for developing or improving casemix models for home care.

## 1 INTRODUCTION

Countries around the world are grappling with the challenge of maintaining a sustainable healthcare system. Ageing populations and the increasing prevalence of chronic disease and multimorbidity are leading to a growing demand for care, pushing healthcare costs steadily higher. As a result, there is increasing pressure to improve the effectiveness and efficiency of the healthcare system (European Commission, 2014; Morris, Powick, & Vetorri, 2016) on the basis of the “Triple Aim”: simultaneously improving care outcomes, improving experiences of care, and lowering the overall per capita cost of care (Berwick, Nolan, & Whittington, 2008).

In most countries, long‐term care accounts for a substantial proportion of total healthcare spending. In 2016, 21% of the healthcare spending in the US was spent on long‐term care (Kaiser Family Foundation, 2017), while in the Netherlands, long‐term care accounted for 27% of total spending on healthcare (Rijksoverheid, 2016). With regard to a sustainable healthcare system, home care is a highly relevant source of long‐term care, because it is known to be more efficient than long‐term institutional care (Genet, Boerma, Kroneman, Hutchinson, & Saltman, 2013). The different types of home‐care services include nursing care (e.g., medication management support or wound care) and personal care (e.g., assistance with bathing).

In most Western countries, home care is funded on a fee‐for‐service basis, but this can create perverse incentives for providers (Miller, 2009). For instance, fee‐for‐service funding is known to stimulate quantity of care rather than quality of care: the more services that home‐care providers deliver, the more money they earn (Koster, Harmsen, & van der Palen, 2015; Miller, 2009). This is inconsistent with recent approaches to home care, which focus on increasing self‐reliance and independence of clients (Tuntland, Aaslund, Espehaug, Forland, & Kjeken, 2015; V&VN, 2014), such as the “Reablement” approach (also known as restorative care). According to reablement, home‐care services should be goal‐oriented, holistic, and person‐centred, taking into account the capabilities of older adults and their social network (Metzelthin, Zijlstra, & Rossum, et al., 2017; Ryburn, Wells, & Foreman, 2009). Furthermore, fee‐for‐service funding creates a higher administrative burden for home‐care providers due to the plethora of administrative requirements and the complexity of funding arrangements (Vereniging van Nederlandse gemeenten (VNG), 2016; V&VN, 2014).

A potential solution that could improve the sustainability of healthcare systems, and in particular home care, would be to implement casemix funding. This would involve categorising clients into homogenous, hierarchical groups according to their actual need for home care, based on an assessment of, for example, their clinical and/or functional status and, in some cases, the level of social support available (Poss, Hirdes, Fries, McKillop, & Chase, 2008). For each of these so‐called casemix groups, a specific budget—in terms of allocated care (funds)—would be determined. Rather than incentivising service volume, casemix funding would incentivise providers to provide needs‐based, high‐quality, and efficient care that focuses on increasing self‐reliance and independence of clients. This would help countries to achieve the Triple Aim (Seow & Sibley, 2014) and it could be a solution to the high administrative burden in home care, simplifying the funding model and using standardised registrations, such as data from nursing classification systems, as a basis (van Rijn, 2016).

Several countries have already successfully developed casemix models for home care, each of them highlighting different casemix groups and including a variety of client characteristics to assess home‐care needs (Elissen, Metzelthin, van den Bulck, Verbeek, & Ruwaard, 2017; Parsons, 2016; Poss et al., 2008). For example, in the US, two casemix models have been developed: Home Health Resource Groups (HHRGs), which is adapted to Medicare reimbursement and uses casemix groups based on the Outcome and Assessment Information Set (OASIS) (Elissen et al., 2017; Poss et al., 2008), and Resource Utilization Groups (version 3) for Home Care (RUG‐III/HC), based on a standardised assessment (RAI) and validated in Canada (Poss et al., 2008). However, due to differences between national healthcare systems, adaptations would likely be necessary to implement existing casemix models in other countries (Genet et al., 2013).

In 2017, on behalf of the Dutch Ministry of Health, Welfare and Sport, the Dutch Healthcare Authority (NZa) initiated a joint venture with knowledge partners to create a knowledge base for the development of a new casemix model for home care in the Netherlands (van Rijn, 2016). Rather than incentivising the volume of care, the new model should incentivise nurses working in home care (further referred to as home‐care nurses) to—based on their professional knowledge and experience—provide high‐quality care that is tailored to clients’ needs.

Home‐care nurses will play a major role in developing the new model, since they have valuable insight into their clients’ needs, and the type and amount of home care required, because they regularly perform home‐care needs assessments. The aim of this survey study was therefore to determine which client characteristics are predictors of clients’ needs for home care, according to home‐care nurses in the Netherlands. These insights are valuable for the development of (casemix groups for) a Dutch home‐care funding model, as well as in other countries that use casemix‐based models to analyse or review their existing funding model for home care.

## 2 METHODS

### 2.1 Study design and respondents

A cross‐sectional survey study was conducted among Dutch home‐care nurses in May 2017. The survey’s target group consisted of approximately 20,000 Dutch home‐care nurses (Bloemendaal, van Essen, Kramer, & van der Windt, 2015; van der Windt & Bloemendaal, 2015), who can be divided into about 9,000 district nurses (bachelor prepared registered nurses, with or without additional postgraduate education, Dutch Qualification Framework (NLQF) level 6) and about 11,000 vocational nurses (vocationally trained registered nurses, NLQF level 4) (Genet et al., 2013). The primary target group for this study was level 6 nurses, since they were assumed to perform home‐care needs assessments in the Netherlands. In practice, vocational nurses are also involved in this task if they meet certain criteria. Vocational nurses were therefore included. The aim was to maximise the response rate within the target group.

### 2.2 Survey development

The content of the survey was based on relevant literature. Seven reports were identified that describe existing casemix models for home care and/or client characteristics that potentially predict home‐care needs. These reports were studied in order to design the survey. One report describes a systematic literature search conducted in 2014 on behalf of the Dutch Ministry of Health, Welfare and Sport, focusing on the client characteristics used in funding models to predict clients’ healthcare needs (Elissen, Struijs, Baan, & Ruwaard, 2015). The other six reports published thereafter, related to home‐care casemix models and/or client characteristics, were sourced from the researchers’ personal network (Elissen et al., 2017; Gupta Strategists, 2016; Koster et al., 2015; Parsons, 2016; Stam & Stadlander, n.d.; V&VN, 2016).

The seven reports identified three home‐care casemix models. In addition to the US models, RUG‐III/HC and HHRG (Elissen et al., 2015), mentioned above, New Zealand’s Home and Community Support Services Case Mix (HCSS CM) was identified. In this model, a brief screening is performed to assign clients to either a complex or a noncomplex casemix group. Next, the clients’ home‐care needs are assessed using the InterRAI Full Assessment or the InterRAI Contact Assessment respectively (InterRAI, 2016; Parsons, 2016). All seven of the reports analysed described client characteristics that were potential predictors of clients’ home‐care needs (Elissen et al., 2017, 2015; Gupta Strategists, 2016; Koster et al., 2015; Parsons, 2016; Stam & Stadlander, n.d.; V&VN, 2016).

Based on the seven reports, client characteristics that potentially predict home‐care needs were extracted. This analysis, taking place in April 2017, resulted in an extended list of 118 client characteristics which were potential predictors of home‐care needs. All characteristics were defined using nursing literature (Gordon, 2014; Herdman, 2014; Herdman & Kamitsuru, 2014; van Achterberg, Bours, & Eliens, 2011, 2012 ). Characteristics were then selected by the researchers based on their potential relevance. The criterion applied was that the characteristic had to be included in at least one casemix model or be mentioned in at least two reports. Where possible, client characteristics were clustered with similar characteristics. Finally, the list was reduced to 35 client characteristics (Table 1) through a consultation process involving multiple stakeholders. Those characteristics were clustered into eight categories: sociodemographic characteristics (*n* = 4); social environmental characteristics (*n* = 3); physical functioning (*n* = 5); daily functioning (*n* = 4); cognitive functioning (*n* = 2); mental functioning (*n* = 4); behaviour (*n* = 6); and health status and services (*n* = 7). All 35 characteristics were redefined and then, including their definitions, incorporated into the survey. The survey was developed in co‐operation with the Dutch Nurses’ Association (V&VN, the sectoral association for nurses and carers in the Netherlands) and the Dutch Healthcare Authority (NZa), and tested and approved by stakeholders from various organisations (i.e., Utrecht University and Utrecht University of Applied Sciences, Tilburg University, the Dutch Society of Community Nurses (NWG) and the Dutch Patient Federation).

**Table 1:** Client characteristics retrieved from available casemix funding models and additional reports

Client characteristic (*N* = 35)	HCSS CM (Parsons et al., 2016)	RUG‐II/HC (Elissen et al., 2014; Pasons et al., 2016; Poss et al., 2008)	HHRG (Elissen et al., 2014)	Elissen et al. (2014)	Parsons et al. (2016)	Gupta Strategists (2016)	Koster et al. (2015)	Elissen et al. (2017)	V&VN (2016)	Stam and Stadlander (in progress)
	Available funding models			Additional reports						
**Socio‐demographic characteristics**										
1. Age				x			x	x	x	x
2. Sex						x	x	x		x
3. Socio‐economic status				x	x	x		x	x	x
4. Area of living				x			x	x	x	x
**Social environmental characteristics**										
5. Composition of household				x	x			x		x
6. Social support	x			x				x	x	x
7. Burden of informal caregiver	x							x	x	x
**Physcial functioning**										
8. Physical functions	x						x	x		x
9. Indoor mobility	x	x	x				x			
10. Outdoor mobility		x					x			
11. Sensory ability			x		x		x	x		x
12. Bladder and bowel continence	x		x				x	x		
**Daily functioning**										
13. ADL functioning	x	x	x	x			x	x		
14. IADL functioning	x	x		x			x	x		
15. Participation in social activities	x						x			
16. Medication management	x	x		x			x	x		x
**Cognitive functioning**										
17. Cognitive functions	x	x	x				x	x		x
18. Awareness of own health issues								x	x	x
**Mental functioning**										
19. Motivation								x	x	
20. Emotional concerns				x				x		x
21. Anxiety								x		x
22. Signs of depression								x		x
**Behaviour**										
23. Lifestyle		x		x			x	x		x
24. Problem behaviour		x	x				x			
25. Resistance to receiving care										x
26. Self‐directing									x	x
27. Self‐management						x		x	x	x
28. Coping							x	x		x
**Health status and services**										
29. Stability	x							x	x	
30. Revalidation phase	x	x					x	x		x
31. Presence of chronic disease		x	x	x			x			x
32. Multi‐morbidity								x	x	x
33. Complications of (chronic) disease	x	x		x						x
34. Terminal phase		x				x	x			
35. Complex or specialized care		x	x		x		x			

### 2.3 Procedure

Home‐care nurses were approached through convenience sampling, as this is an efficient method to reach a large population of home‐care nurses across the country. The survey was posted online on the website of V&VN on May 3, 2017. On May 4, 2017 the survey link was also publicised in the newsletter of V&VN. Two weeks later (May 18, 2017), a reminder was sent with the subsequent V&VN newsletter. Meanwhile, home‐care nurses were approached via the researchers’ personal network and the stakeholders involved, via Twitter and LinkedIn, via internal communication channels of healthcare organisations, and through articles posted on Skipr, a Dutch healthcare news website (www.skipr.nl), and the NZa website. The survey was closed after 21 days (on May 23, 2017). Only completed surveys were included in the analyses. Respondents completed the survey anonymously. Participation in the survey was voluntary. Information on the reason, goal, contents and development of the survey, and contact information were included in the survey’s introduction. Respondents were not asked to declare informed consent since no approval is needed according to the Dutch Medical Research (Human Subjects) Act (WMO).

### 2.4 Measures

The survey consisted of four sections: (a) background characteristics of the respondent, (b) 35 client characteristics which were to be scored on their relevance to predicting the clients’ needs for home care, (c) an opportunity to name, define, and score up to two missing client characteristics; and (d) a request to choose and rank the top five client characteristics.

The following background characteristics were collected on the respondents: sex, age, education, years of working experience in home care, function (i.e., district nurse or vocational nurse), whether the respondent conducts needs assessments or not, whether the respondent works as a generalist and/or specialist, working area (i.e., zip code of the area in which the respondent mainly works), whether the respondent is currently working in home care or not, and whether the respondent is member of V&VN.

The relevance of each of the 35 potential client characteristics to indicate home‐care needs was scored on a 9‐point Likert scale from 1 (totally irrelevant) to 9 (extremely relevant). Respondents were asked to score characteristics independently of possible interaction with other client characteristics.

If the respondent thought a relevant client characteristic was missing from the survey, up to two client characteristics could be added. Missing characteristics were named, defined, and scored using the same 9‐point Likert scale.

Finally, respondents chose and ranked the top five characteristics from the entire selection available, that is, 35 characteristics included in the survey plus the one or two that they may have added.

### 2.5 Data analysis

The background characteristics of the sample were analysed using descriptive statistics (i.e., frequencies, percentages, means, and minimum and maximum scores). To assess the relevance of the 35 client characteristics and determine the consensus of opinions among the respondents regarding relevance, medians and interquartile ranges (IQR) were calculated. IQR was used to define the degree of consensus between respondents. In line with previous research (Elissen et al., 2017, 2015), consensus about relevance was defined as a median between 7 and 9, combined with an IQR ≤ 1.5. A median between 1 and 3 combined with IQR ≤ 1.5 meant consensus for irrelevance. All other possibilities with a median between 4 and 6 or IQR > 1.5 were defined as uncertain.

In a sensitivity analysis, vocational nurses who do not perform home‐care needs assessments were excluded, since they could have less insight into client characteristics that predict home‐care needs.

Client characteristics added by the respondents were analysed by listing these answers and clustering similar characteristics based on the definitions provided. Missing characteristics overlapping with 1 or more of the 35 characteristics from the survey were excluded from further analysis. The remaining characteristics were ordered according to the frequency with which they were added by respondents. Missing client characteristics were only included for further analysis if they were mentioned by ≥5 respondents.

The ranked top five client characteristics received a score ranging from 1 (least relevant out of the ranked five) to 5 (most relevant). The scores were added, resulting in a sum score for each individual characteristic that indicated the characteristic’s ranking within the total set of characteristics, based on the rankings of all respondents.

## 3 RESULTS

### 3.1 Respondents

A total of 1,007 home‐care nurses completed the online survey, which corresponds with 5% of the total number of Dutch home‐care nurses (Bloemendaal et al., 2015; van der Windt & Bloemendaal, 2015). Table 2 shows the background characteristics of the respondents. Most were district nurses (*n* = 757, 75%); years of working experience ranged from 0 to 44 years, with an average of 10 years. Furthermore, all 12 provinces of the Netherlands were represented, with between 13 and 205 respondents per province.

**Table 2:** Background characteristics of respondents (*N* = 1,007)

	*N* (%)	Mean	Minimum	Maximum
Sex				
Female	948 (94.1)			
Male	59 (5.9)			
Age				
All ages		40.2	19	66
≤25 years	151 (15.0)			
26–40 years	364 (36.1)			
41–55 years	341 (33.9)			
≥56 years	151 (15.0)			
Education				
High school or Secondary Vocational Education (SVE)	203 (20.2)			
University of Applied Sciences (UAS)	748 (74.3)			
University	42 (4.2)			
Other	14 (1.4)			
Years of working experience in home care				
All		10.0	0	44
≤2 years	162 (16.1)			
3–7 years	364 (36.1)			
8–19 years	313 (31.1)			
≥20 years	168 (16.7)			
Function				
District nurse	757 (75.2)			
Vocational nurse	202 (20.1)			
Other	48 (4.8)			
Conducting home‐care needs assessments				
Yes	854 (84.8)			
No^a^	153 (15.2)			
Generalist or specialist				
Generalist	832 (82.6)			
Specialist	62 (6.2)			
Generalist and specialist	113 (11.2)			
Currently working as home‐care nurse				
Yes	972 (96.5)			
No	35 (3.5)			
Membership V&VN				
Membership V&VN	762 (75.7)			
No membership V&VN	245 (24.3)			

a 110 vocational nurses (54% of the vocational nurses; 11% of all respondents) do not conduct home‐care needs assessments.

### 3.2 Relevance of and consensus on the 35 client characteristics

Table 3 presents the medians and IQRs for each individual client characteristic. Thirty client characteristics achieved a median score of ≥ 7. A consensus on relevance was found for 15 of these characteristics (IQR ≤ 1.5). The highest degree of consensus on relevance was achieved by the characteristic “terminal phase” (median = 9 and IQR = 1). The relevance of the remaining 20 characteristics was uncertain: in 19 cases, this was due to both the median score between 4 and 7 and the lack of consensus on relevance (IQR > 1.5); in one case this was due to lack of consensus on irrelevance (median score ≤ 3 and IQR > 1.5). These 20 uncertain client characteristics included all characteristics in the categories of “sociodemographic characteristics” (*n* = 4) and “mental functioning” (*n* = 4), and most characteristics in the category of “daily functioning” (*n* = 3). Furthermore, there was no consensus on any client characteristic being irrelevant (median ≤ 3 and IQR ≤ 1.5).

**Table 3:** Relevance of client characteristics based on median and IQR

	Median	IQR (boundaries)
Relevant client characteristics (*N* = 15)		
Terminal phase	9	1 (8–9)
Complex or specialised care	8	1 (8–9)
Social support	8	1 (7–8)
Burden of informal caregiver	8	1 (7–8)
Physical functions	8	1 (7–8)
Indoor mobility	8	1 (7–8)
Medication management	8	1 (7–8)
Awareness of own health issues	8	1 (7–8)
Self‐directing	8	1 (7–8)
Self‐management	8	1 (7–8)
Presence of chronic disease	8	1 (7–8)
Complications of (chronic) disease	8	1 (7–8)
Revalidation phase	7	1 (7–8)
Sensory ability	7	1 (6–7)
Composition of household	7	1 (6–7)
Uncertain client characteristics (*N* = 20)		
ADL functioning	8	2 (7–9)
Cognitive functions	8	2 (7–9)
Multimorbidity	8	2 (7–9)
Age	7	2 (6–8)
Bladder and bowel continence	7	2 (6–8)
IADL functioning	7	2 (6–8)
Motivation	7	2 (6–8)
Emotional concerns	7	2 (6–8)
Anxiety	7	2 (6–8)
Signs of depression	7	2 (6–8)
Lifestyle	7	2 (6–8)
Problem behaviour	7	2 (6–8)
Resistance to receiving care	7	2 (6–8)
Coping	7	2 (6–8)
Stability	7	2 (6–8)
Socioeconomic status	6	2 (5–7)
Area of living	6	2 (5–7)
Outdoor mobility	6	2 (5–7)
Participation in social activities	6	2 (5–7)
Sex	3	4 (1–5)
Irrelevant client characteristics (*N* = 0)		
None		

No respondent scored all the characteristics as irrelevant (score ≤ 3). Fifteen respondents (2%) scored all client characteristics as relevant (score ≥ 7), one of whom (0%) scored all characteristics with a score of 9.

The results of a sensitivity analysis showed that results of the survey did not differ when vocational nurses who do not perform the assessment (*n* = 110) were excluded, except for a small difference in IQR for “revalidation phase”: For the total sample, IQR was 2, while when excluding the described group, IQR was 1.

### 3.3 Missing client characteristics

In total, 62 missing client characteristics were mentioned by 112 respondents (11%). Most of these (60%) overlapped with 1 or more of the proposed 35 characteristics and were therefore excluded. The remaining 25 missing client characteristics were mentioned by a minimum of 1 and a maximum of 10 respondents, of which eight characteristics were mentioned by ≥ 5 respondents. The most frequently mentioned missing client characteristic was “living situation” (*n* = 10), meaning the safety, hygiene, or liveability of the client’s housing, which could be placed into the category “social environmental characteristics.” Other missing characteristics mentioned by five to nine respondents related to the categories “sociodemographic characteristics” (i.e., financial situation and ethnicity), “cognitive functioning” (i.e., communication (skills)), “mental functioning” (i.e., sense of coherence and loneliness), “behaviour” (i.e., nutrition), and “health status and services” (i.e., mental illnesses and multidisciplinary care).

### 3.4 Ranking client characteristics

Based on the sum scores for the respondents’ rankings, “ADL functioning” was the most relevant client characteristic for predicting the clients’ home‐care needs. Among respondents, 45% chose “ADL functioning” as one of the top five ranked characteristics. “Outdoor mobility” was ranked least relevant. Table 4 represents the ranking of all 35 client characteristics.

**Table 4:** Ranking client characteristics based on sum of scores for the top five client characteristics

Overall rank	Client characteristic	Chosen as one of the top five characteristics *N* (%)	Rank 1 score 5 *N* (%)^a^	Rank 2 score 4 *N* (%)^a^	Rank 3 score 3 *N* (%)^a^	Rank 4 score 2 *N* (%)^a^	Rank 5 score 1 *N* (%)^a^	Sum total score
1	ADL functioning	451 (44.8)	126 (27.9)	113 (25.1)	84 (18.6)	80 (17.7)	48 (10.6)	1,542
2	Terminal phase	392 (38.9)	130 (12.9)	62 (15.8)	44 (11.2)	56 (14.3)	100 (25.5)	1,242
3	Cognitive functions	389 (38.6)	66 (17.0)	101 (26.0)	107 (27.5)	71 (18.3)	44 (11.3)	1,241
4	Physical functions	278 (27.6)	99 (35.6)	67 (24.1)	43 (15.5)	39 (14.0)	30 (10.8)	1,000
5	Social support	342 (34.0)	40 (11.7)	69 (20.2)	89 (26.0)	80 (23.4)	64 (18.7)	967
6	Burden of informal care‐giver	330 (32.8)	28 (8.5)	68 (20.6)	95 (28.8)	83 (25.2)	56 (17.0)	919
7	Complex or specialised care	302 (30.0)	69 (22.8)	59 (19.5)	50 (16.6)	52 (17.2)	72 (23.8)	907
8	Multimorbidity	299 (29.7)	52 (17.4)	65 (21.7)	61 (20.4)	62 (20.7)	59 (19.7)	886
9	Self‐directing	251 (24.9)	91 (36.3)	45 (17.9)	41 (16.3)	41 (16.3)	33 (13.1)	873
10	Self‐management	245 (24.3)	67 (27.3)	56 (22.9)	48 (19.6)	32 (13.1)	42 (17.1)	809
11	Presence of chronic disease	216 (21.4)	35 (16.2)	40 (18.5)	50 (23.1)	44 (20.4)	47 (21.8)	620
12	Medication management	206 (20.5)	12 (5.8)	32 (15.5)	52 (25.2)	61 (29.6)	49 (23.8)	515
13	Complications of (chronic) disease	189 (18.8)	21 (11.1)	32 (16.9)	38 (20.1)	55 (29.1)	43 (22.8)	500
14	Awareness of own health issues	153 (15.2)	31 (20.3)	30 (19.6)	39 (25.5)	34 (22.2)	19 (12.4)	479
15	Age	124 (12.3)	40 (32.3)	19 (15.3)	15 (12.1)	14 (11.3)	36 (29.0)	385
16	Motivation	82 (8.1)	11 (13.4)	20 (24.4)	16 (19.5)	25 (30.5)	10 (12.2)	243
17	Socioeconomic status	90 (8.9)	12 (13.3)	19 (21.1)	15 (16.7)	12 (13.3)	32 (35.6)	237
18	Coping	90 (8.9)	10 (11.1)	11 (12.2)	16 (17.8)	23 (25.6)	30 (33.3)	218
19	Lifestyle	77 (7.6)	10 (13.0)	12 (15.6)	17 (22.1)	18 (23.4)	20 (26.0)	205
20	Indoor mobility	78 (7.7)	7 (9.0)	15 (19.2)	18 (23.1)	17 (21.8)	21 (26.9)	204
21	Resistance to receiving care	66 (6.6)	8 (12.1)	6 (9.1)	10 (15.2)	24 (36.4)	18 (27.3)	160
22	Composition of household	53 (5.3)	4 (7.5)	14 (26.4)	10 (18.9)	11 (20.8)	14 (26.4)	142
23	Problem behaviour	51 (5.1)	3 (5.9)	7 (13.7)	8 (15.7)	13 (25.5)	20 (39.2)	113
24	Bladder and bowel continence	49 (4.9)	2 (4.1)	*8 * *(* *16.3)*	7 (14.3)	10 (20.4)	22 (44.9)	105
25	IADL functioning	25 (2.5)	4 (16.0)	4 (16.0)	6 (24.0)	7 (28.0)	4 (16.0)	72
26	Stability	27 (2.7)	3 (11.1)	6 (22.2)	1 (3.7)	9 (33.3)	8 (29.6)	68
27	Area of living	29 (2.9)	3 (10.3)	4 (13.8)	5 (17.2)	2 (6.9)	15 (51.7)	65
28	Emotional concerns	24 (2.4)	2 (8.3)	5 (20.8)	3 (12.5)	6 (25.0)	8 (33.3)	59
29	Signs of depression	20 (2.0)	2 (10.0)	4 (20.0)	3 (15.0)	4 (20.0)	7 (35.0)	50
30	Anxiety	19 (1.9)	2 (10.5)	*2 * *(* *10.5)*	3 (15.8)	8 (42.1)	4 (21.1)	47
31	Participation in social activities	18 (1.8)	0 (0.0)	3 (16.7)	3 (16.7)	4 (22.2)	8 (44.4)	37
32	Revalidation phase	17 (1.7)	0 (0.0)	1 (5.9)	4 (23.5)	3 (17.6)	9 (52.9)	31
33	Sensory ability	7 (0.7)	2 (28.6)	1 (14.3)	1 (14.3)	2 (28.6)	1 (14.3)	22
34	Sex	3 (0.3)	1 (33.3)	1 (33.3)	0 (0.0)	0 (0.0)	1 (33.3)	10
35	Outdoor mobility	1 (0.1)	0 (0.0)	1 (100.0)	0 (0.0)	0 (0.0)	0 (0.0)	4

Example calculation for the ranking of ADL functioning: The first column is the percentage of respondents who ranked ADL functioning in the top five characteristics, that is, 45% of the respondents. The following five columns explain how many respondents ranked ADL functioning in, respectively, rank 1 (i.e., 27.9% of those 45%), rank 2 (i.e. 25.1% of those 45%), rank 3 (i.e., 18.6% of those 45%), rank 4 (17.7% of those 45%) and rank 5 (i.e., 10.6% of those 45%). Finally, the total sum score was calculated to determine the overall rank of ADL functioning among all 35 characteristics by the following calculation: (126 × score 5) + (113 × score 4) + (84 × score 3) + (80 × score 2) + (48 × score 1) = 1,542. The characteristic with the highest total sum score was placed on overall rank 1, and the lowest on overall rank 35.

a Percentages relate to *N* of “chosen as one of the top five characteristics.”

## 4 DISCUSSION

The aim of this study was to determine which client characteristics predict clients’ needs for home care according to Dutch home‐care nurses. To achieve this aim, based on a review of international literature, 35 potentially relevant client characteristics were included in a cross‐sectional, online survey. A total of 1,007 nurses completed the survey (i.e., 5% of Dutch home‐care nurses). There was a consensus among the respondents regarding the relevance of 15 client characteristics for predicting clients’ needs for home care.

Across the client characteristics included in the survey, higher median scores for relevance were associated with lower IQRs. Hence, it seems that a stronger consensus exists among nurses regarding those characteristics that are generally considered more relevant, such as “terminal phase” and “indoor mobility.” Moreover, this confirms that characteristics on which there was uncertainty among home‐care nurses (median < 7 and/or IQR > 1.5), are indeed uncertain. However, there were three notable exceptions to this: the characteristics “ADL functioning,” “cognitive functioning,” and “multimorbidity.” Although there was insufficient consensus among the nurses on the relevance of these characteristics (IQR > 1.5), they attained among the highest individual scores for relevance (medians of 8) and were ranked in the overall top 10 of the most relevant factors (rank 1, 3, and 8 respectively). Also, “ADL functioning” and “cognitive functioning” were the only client characteristics included in all casemix models consulted (Elissen et al., 2015; New Zealand Productivity Commission, 2015; Parsons, 2016). Both are widely considered as important predictors of home‐care needs, and are therefore expected to support efficient planning and organisation of home care (Døhl, Garåsen, Kalseth, & Magnussen, 2016). One possible explanation for the contradictory findings could be differing interpretations of these characteristics by home‐care nurses, in particular regarding the causal relationship with home‐care needs. For example, some nurses may consider limited “ADL functioning” to be a direct and important cause of home‐care needs, and, as such, score and rank “ADL functioning” highly. Other nurses may have viewed the same limitation not as a direct cause, but as a symptom of a more important, underlying problem (e.g., cognitive limitations) resulting in a need for home care (Døhl et al., 2016). As such they could have scored and ranked “ADL functioning” lower. Also, the survey only measured the relevance of client characteristics individually, while in practice combinations of characteristics may determine home‐care needs. Additional qualitative research, such as in‐depth interviews with home‐care nurses, would provide further insight into the nurses’ interpretations and considerations, in order to identify the reasons for these contradictions.

The 15 consensually relevant client characteristics to predicting home‐care needs identified in this study relate to both biomedical determinants, such as “terminal phase” and “physical functions,” and psychosocial determinants of health, such as “social support” and “self‐management.” The nurses therefore seem to believe that the biopsychosocial perspective (van Tilburg, 2016) is relevant when assessing clients’ needs. This is consistent with the nature of the work done by home‐care nurses, as stated in their professional profile: home‐care nurses should be able to handle increasing complexity of clients by incorporating a holistic, biopsychosocial perspective (de Bont, van Haaren, Rosendal, & Wigboldus, 2012; Schuurmans, Lambregts, Grotendorst, & van Merwijk, 2012). However, a biopsychosocial perspective has not yet been incorporated into most existing casemix models. Four of the relevant characteristics (27%) are psychosocial characteristics and did not appear in any of the models at all: “composition of household,” “awareness of own health issues,” “self‐directing,” and “self‐management.” Most current casemix models were developed based on a more biomedical model of health (Parsons et al., 2018; Wade & Halligan, 2017). According to previous research, this is suitable when determining casemix in an inpatient setting, since biomedical characteristics—such as a diagnosis—are accurate predictors of service need in, for example, a nursing home, as well as based on valid, reliable, and available data (Fries et al., 1994; Laporte, Croxford, & Coyte, 2007; Parsons et al., 2018). However, determining casemix in the community, including contextual factors—such as health status of the informal caregiver—provides a more reliable representation of the client’s care needs (Björkgren, Fries, & Shugarman, 2000; Parsons et al., 2018). However, including psychosocial data in a home‐care funding model is viewed as a challenge (Elissen et al., 2015), since most routinely collected data concern biomedical determinants of health (Björkgren et al., 2000; Elissen et al., 2015; Laporte et al., 2007).

This study has certain strengths and limitations. First, it is unknown how many V&VN members met the inclusion criteria. Also, the exact number of Dutch home‐care nurses is uncertain, since different sources report different numbers, which makes it difficult to determine a precise response rate. Yet, based on an estimated total population of 20,000 home‐care nurses (Bloemendaal et al., 2015; van der Windt & Bloemendaal, 2015), we have a response rate of 5%, which is considerable. Furthermore, background characteristics of the respondents concerning sex (i.e., 94% female) and age (i.e., mean age of 40 years) only slightly deviate from the available population characteristics (i.e., approximately 92% female; approximate mean age of 44 years) (Arbeidsmarkt Zorg en Welzijn, 2015) and all provinces of the Netherlands were represented. Therefore, the sample is considered as being representative. Respondents who completed the questionnaire had no missing values, as they were obliged to fill in all questions. However, there are no data about respondents who did not complete the survey, as only completed surveys were saved and included in the study. According to previous research on large‐scale web‐based surveys, about 10% of respondents who start a survey quits nearly instantaneously, with an additional 2% dropout per 100 survey items (Hoerger, 2010). Given the size and diversity of this survey sample, there is no reason to assume that dropouts are not at random. A strength of this study is the comprehensive, systematic selection of client characteristics for the survey. A wide range of reports and several existing casemix models were screened for client characteristics. Although there will always be a possibility that relevant characteristics were overlooked due to unknown or unpublished studies, the low maximum frequency (*n* = 10) with which respondents added characteristics suggests that the survey was relatively comprehensive. Furthermore, defining client characteristics using nursing literature led to unambiguous interpretation, in line with the nursing profession, on the meaning of each characteristic.

This study aimed at exploring the view of home‐care nurses in general. Results were compared for one subgroup, that is, by performing a sensitivity analysis for vocational nurses who do not perform home‐care needs assessments. As a subsequent step, a qualitative study is planned to get more in‐depth information if and why these findings would differ for relevant (other) subgroups, by, for example, looking at available resources in the community, or rural versus urban working areas.

As far as we are aware, this survey study among Dutch home‐care nurses is one of the first attempts to utilise nurses’ professional knowledge and experience in order to develop a casemix model. The involvement of home‐care nurses is expected to help in the development of a funding model that is both robust and suitable for clinical practice, and maximise trust and support during implementation. Besides continuous involvement of nurses, quantitative research is necessary to collect objective information concerning the coherence and predictability of (combinations of) relevant client characteristics and home‐care needs. It is therefore recommended to examine the client data routinely collected, bearing in mind the paradigm shift in home care over recent years and its effect on reported data. Data from various sources, such as health and social care providers and municipalities, should be included to compensate for the lack of psychosocial data.

## 5 CONCLUSION

Based on a review of relevant literature, a comprehensive set of client characteristics was presented to home‐care nurses in a survey to determine their relevance to predicting clients’ home‐care needs. Although a strong consensus was revealed concerning the relevance of some characteristics, discrepancies were also identified between responses, possibly due to differences in interpretation. According to the respondents, client characteristics that are relevant to predicting home‐care needs are of both biomedical and psychosocial nature. However, even though incorporating a biopsychosocial perspective into a funding model could provide the right incentives to work towards the Triple Aim, current home‐care funding models often omit psychosocial determinants of health, making the funding model being less in line with clinical practice. To incorporate the biopsychosocial perspective, close collaboration between clinical practice, policy development, and science—by combining connected clients’ data from different sources with the knowledge and experience of home‐care nurses, for example—is necessary. This could improve both existing (casemix) funding models and facilitate the development of new models.

## CONFLICT OF INTEREST

The authors declare that they have no conflict of interest.
